# Supplementary material for: Trends in Antituberculosis Drug Resistance and Associated Factors: A 31-Year Observational Study at a Tertiary Hospital in Barcelona
Source: Antibiotics (Basel). 2025 Aug 30;14(9):875. doi: 10.3390/antibiotics14090875 (PMC12466672; doi:10.3390/antibiotics14090875)
Supplement: Supplementary file 1 [file antibiotics-14-00875-s001.zip › antibiotics-3823522-supplementary.pdf]

antibiotics

MDPI

Supplementary Materials: 1

The following supporting information can be downloaded at: https://www.mdpi.com/article/doi/s1, 2

Table S1. Distribution according to drug resistance, case classification and origin. 3

	NEW CASES			PREVIOUSLY TREATED CASES		
	TOTAL (N=2.448)	Spain born (N=1.549)	Foreign Born (N=640)	TOTAL (N=259)	Spain born (N=216)	Foreign born (N=43)
	No. (%)	No. (%)	No. (%)	No. (%)	No. (%)	No. (%)
Resistance [1]						
• H	139 (6.3)	66 (4.3)	73 (11.4)	51 (19.7)	32 (14.8)	19 (44.2)
• R	35 (1.6)	15 (1.0)	20 (3.1)	24 (9.3)	15 (7.0)	9 (20.9)
• E	20 (0.9)	9 (0.6)	11 (1.7)	4 (1.6)	3 (1.4)	1 (2.3)
• Z	51 (2.3)	23 (1.5)	28 (4.4)	7 (2.7)	2 (0.9)	5 (11.6)
• S	73 (8.8)	24 (4.9)	49 (14.5)	13 (16.7)	8 (15.4)	5 (19.2)
Monoresistance						
• H	81 (3.7)	42 (2.7)	39 (6.1)	21 (8.1)	13 (6.1)	8 (18.6)
• R	3 (0.1)	1 (0.1)	2 (0.3)	0 (0.0)	0 (0.0)	0 (0.0)
• E	8 (0.4)	4 (0.3)	4 (0.6)	0 (0.0)	0 (0.0)	0 (0.0)
• Z	29 (1.3)	14 (0.9)	15 (2.3)	0 (0.0)	0 (0.0)	0 (0.0)
• S	63 (2.9)	21 (1.4)	42 (6.6)	3 (1.2)	2 (0.9)	1 (2.3)
Multiresistance						
• H+R	11 (0.5)	4 (0.3)	7 (1.1)	12 (4.7)	9 (4.2)	3 (7.0)
• H+R+E	1 (0.1)	1 (0.1)	0 (0.0)	1 (0.4)	1 (0.5)	0 (0.0)
• H+R+Z	4 (0.2)	3 (0.2)	1 (0.2)	5 (1.9)	2 (0.9)	3 (7.0)
• H+R+S	3 (0.1)	1 (0.1)	2 (0.3)	3 (1.2)	2 (0.9)	1 (2.3)
• H+R+E+Z	2 (0.1)	2 (0.1)	0 (0.0)	1 (0.4)	0 (0.0)	1 (2.3)
• H+R+E+S	2 (0.1)	0 (0.0)	2 (0.3)	1 (0.4)	1 (0.5)	0 (0.0)
• H+R+Z+S	4 (0.2)	2 (0.1)	2 (0.4)	1 (0.4)	0 (0.0)	1 (2.3)
• H+R+E+Z+S	5 (0.2)	1 (0.1)	4 (0.6)	0 (0.0)	0 (0.0)	0 (0.0)
Polyresistance						
• S+Z	3 (0.1)	0 (0.0)	3 (0.5)			
• H+E	1 (0.1)	1 (0.1)	0 (0.0)	0 (0.0)	0 (0.0)	0 (0.0)
• H+Z	3 (0.1)	1 (0.1)	2 (0.3)	0 (0.0)	0 (0.0)	0 (0.0)
• H+S	20 (0.9)	8 (0.5)	12 (1.9)	5 (1.9)	3 (1.4)	2 (4.7)
• H+S+Z	1 (0.1)	0 (0.0)	1 (0.2)	0 (0.0)	0 (0.0)	0 (0.0)
• H+E+S	1 (0.1)	0 (0.0)	1 (0.2)	1 (0.4)	1 (0.5)	0 (0.0)

E: Ethambutol; H: isoniazid; Z: Pyrazinamide R: Rifampicin; S: Streptomycin. 1 including monoresistant, multi- 4
resistant and poliresistant isolate 5

Table S2. Streptomycin resistance distribution by period, level and origin.

ORIGIN	SPAIN						LATIN AMERICA						ASIA						AFRICA						EUROPE (other than Spain)					
PERIOD	1991-2000		2001-2022		TOTAL		1991-2000		2001-2022		TOTAL		1991-2000		2001-2022		TOTAL		1991-2000		2001-2022		TOTAL		1991 - 2000		2001-2022		TOTAL	
TOTAL CASES (n)	1,130		635		1,765		77		229		306		37		140		177		46		90		136		11		53		64	
	N	%	n	%	n	%	n	%	n	%	n	%	n	%	n	%	n	%	n	%	n	%	n	%	n	%	n	%	n	%
Total resistance	13	*1.2*	19	*3.0*	32	*1.8*	3	*3.9*	18	*7.9*	21	*6.9*	3	*8.1*	10	*7.1*	13	*7.3*	1	*2.2*	11	*12.2*	12	*8.8*	0	-	8	*15.1*	8	*12.5*
Low-level resistance (1mg/L) [1]	3	*0.3*	7	*1.1*	10	*0.6*	1	*1.3*	11	*4.8*	12	*3.9*	0	-	7	*5.0*	7	*4.0*	0	*0.0*	7	*7.8*	7	*5.1*	0	-	3	*5.7*	3	*4.7*
High-level resistance (4-6mg/L) [1]	10	*0.9*	12	*1.9*	22	*1.2*	2	*2.6*	7	*3.1*	9	*2.9*	3	*8.1*	3	*2.1*	6	*3.4*	1	*2.2*	4	*4.4*	5	*3.7*	0	-	5	*9.4*	5	*7.8*

 https://doi.org/10.3390/10.3390/antibiotics14090875
